# The Principle of Cortical Development and Evolution

**DOI:** 10.1007/s12264-024-01259-2

**Published:** 2024-07-18

**Authors:** Zhengang Yang

**Affiliations:** State Key Laboratory of Medical Neurobiology and MOE Frontiers Center for Brain Science, Department of Neurology, Institutes of Brain Science, Zhongshan Hospital, Fudan University, Shanghai, 200032 China

**Keywords:** Radial glia, Cortical neurogenesis, Cortical gliogenesis, Cortical expansion, Cortical evolution, FGF-ERK signaling, SHH signaling, BMP7, Interneuron, Human-specific gene

## Abstract

Human’s robust cognitive abilities, including creativity and language, are made possible, at least in large part, by evolutionary changes made to the cerebral cortex. This paper reviews the biology and evolution of mammalian cortical radial glial cells (primary neural stem cells) and introduces the concept that a genetically step wise process, based on a core molecular pathway already in use, is the evolutionary process that has molded cortical neurogenesis. The core mechanism, which has been identified in our recent studies, is the extracellular signal-regulated kinase (ERK)-bone morphogenic protein 7 (BMP7)-GLI3 repressor form (GLI3R)-sonic hedgehog (SHH) positive feedback loop. Additionally, I propose that the molecular basis for cortical evolutionary dwarfism, exemplified by the lissencephalic mouse which originated from a larger gyrencephalic ancestor, is an increase in SHH signaling in radial glia, that antagonizes ERK-BMP7 signaling. Finally, I propose that: (1) SHH signaling is not a key regulator of primate cortical expansion and folding; (2) human cortical radial glial cells do not generate neocortical interneurons; (3) human-specific genes may not be essential for most cortical expansion. I hope this review assists colleagues in the field, guiding research to address gaps in our understanding of cortical development and evolution.

## Introduction

What makes us human? The answer lies in our cerebral cortex, as proposed by Pasko Rakic in 2006: “Within the past 125 years, we have witnessed great strides in understanding the development and evolution of the cerebral cortex, arguably the structure that makes us human” [1]. The cerebral cortex is the biological substrate of our unique cognitive abilities, including memory, complex reasoning, and advanced language.

The embryonic development of the vertebrate forebrain is guided by signals from three major patterning centers: a rostral patterning center that secretes fibroblast growth factors (FGFs), a caudodorsal center that secretes Wingless/Int proteins (WNTs) and bone morphogenetic proteins (BMPs), and a ventral center that secretes sonic hedgehog (SHH) [2–14]. These distinct signaling centers instruct the regional development of the telencephalon along the dorsoventral and rostrocaudal axes. Notably, recent studies have revealed that the topology of patterning signaling centers in the developing human telencephalon is similar to that in the mouse and ferret [15–19].

The cerebral cortex is derived from the dorsal telencephalon, or pallium, which includes the ventral, lateral, dorsal, and medial pallium [20, 21]. The WNT signaling pathway plays a major role in establishing the neurogenic program in the pallium [22–25]. It is believed that BMP and WNT signaling work together in early pallial (hippocampal and cortical) neuroepithelial cells to regulate their quiescence-proliferation balance [26–30]. WNT signaling is gradually downregulated in cortical radial glial (RG) cells after neurogenesis occurs, but WNT response transcription factor genes *Pax6, Dmarta2*, *Emx2*, and *Lhx2* in the cortical RG cells continue to protect and promote the cortical neurogenic program [22, 31–34]. Cortical patterning and neurogenesis are also protected by the GLI3 repressor form (GLI3R)-mediated suppression from the ventralizing effects of SHH and FGF signaling [35–42].

**Fig. 1:**
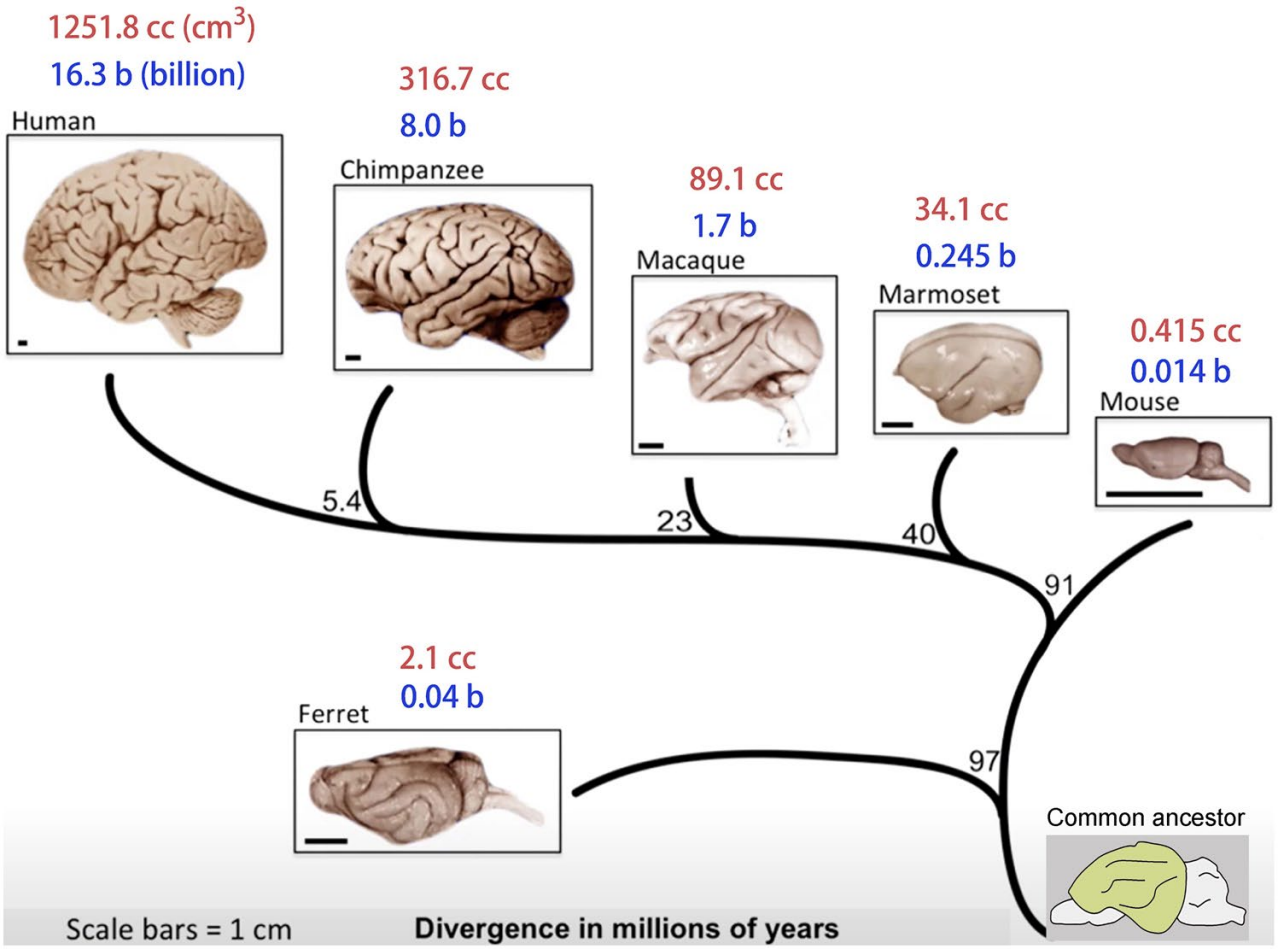
Mammalian cortical expansion and evolution. Mammalian cortical neuron numbers range from 0.014 billion in mice to 16.3 billion in humans. Brain volumes range from 0.4 cc in mice to 1250 cc in humans. I suggest that there is a fundamental conserved genetic/biochemical engine that drives the increase in the number of cortical neurons, which further drives cortical size and brain volume expansion during evolution. Note that the mouse is lissencephalic but originated from a larger and gyrencephalic ancestor, as gyrencephalic ferrets separated before mice from the human phylogenetic tree.

A distinct feature of mammals is their six-layered neocortex (new cortex), which is a specific feature of all mammals. In humans, ~90% of the cerebral cortex is neocortex. Over the course of evolution, the human cerebral cortex attained the largest number of neurons among primates (16.3 billion), which is thought to be the dominant contributor to underlying human intelligence. In contrast, the chimpanzee cerebral cortex has 8 billion neurons, the macaque cortex (old world monkey) has about 1.7 billion, the marmoset (new world monkey) cortex has 0.245 billion, the ferret cortex has 0.04 billion, and the mouse cortex has only 0.014 billion neurons [43–46] (Fig.1). Therefore, to be human, an essential step is to generate 16.3 billion cortical neurons.

## Mouse and Human Cortical Radial Glial Cell Lineage Progression

**Fig. 2:**
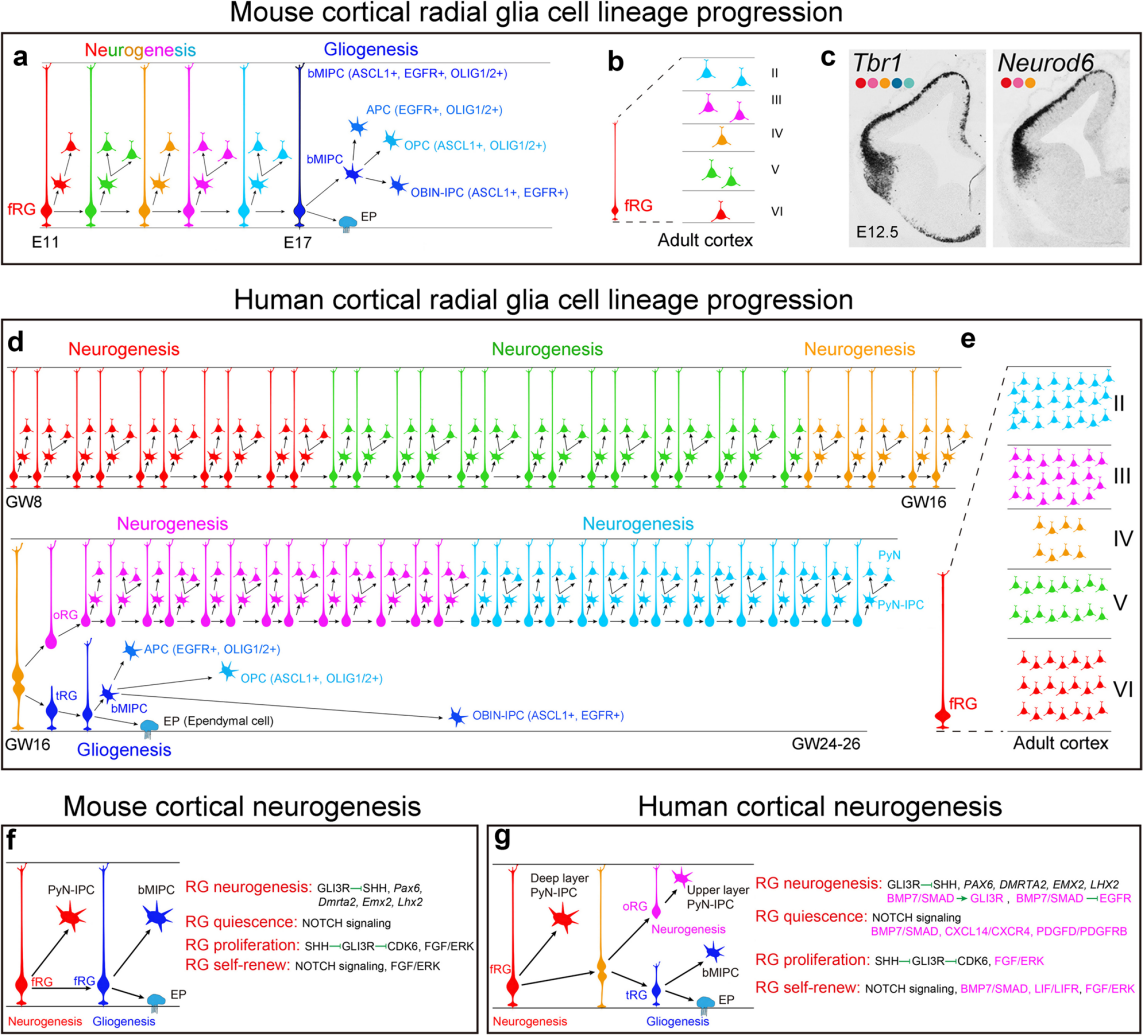
Mouse and human cortical RG cell lineage progression. **a**, **b** Mouse cortical neurogenesis and gliogenesis. Upon entry into the neurogenic phase, a single mouse cortical fRG cell may divide 5-6 times and generate PyN-IPCs, which produce 8–9 PyNs distributed in both deep and upper layers. Cortical gliogenesis occurs after neurogenesis. At the gliogenic stage, mouse fRG cells first generate bMIPCs, which then give rise to cortical APCs, OPCs, and OBIN-IPCs. **c** Mammalian cortical PyNs are first generated in the ventral and lateral pallium, followed by the dorsal and medial pallium (cortex). *Tbr1* and *Neurod6* mRNA in situ hybridization on mouse brain sections at E12.5. Images are reprinted with permission from Moreau *et al.*, 2021 [92]. **d** Human cortical neurogenesis and gliogenesis. I propose that a single human cortical neurogenic fRG cell may divide ~30 times and generate PyN-IPCs, and these PyN-IPCs may produce 30-45 PyNs distributed in deep layers. Around GW16, fRG cells give rise to tRG and oRG cells. A single human oRG cell in the lateral OSVZ may divide ~30 times and generate PyN-IPCs, and these PyN-IPCs may produce 30-45 PyNs distributed in upper layers. Human tRG cells undergo a neurogenesis-to-gliogenesis switch and generate bMIPCs, which divide several times and give rise to cortical APCs, OPCs, and OBIN-IPCs. **e** A single human cortical fRG cell may generate 60-90 PyNs. **f**, **g** Signaling pathways and transcription factors regulate mouse and human cortical RG cell neurogenesis, quiescence, proliferation, and self-renewal. Those signals that are stronger in human cortical RG cells, than in mice, are highlighted in magenta.

Mammalian cortical RG cells, known as cortical primary neural stem cells (NSCs), are the source of all cortical glutamatergic pyramidal neurons (PyNs), astrocytes, and the vast majority of oligodendrocytes [47–53] (Fig. 2a). During cortical development, cortical NSCs in mice primarily have one type of RG cell, known as full-span radial glial (fRG) cells [54, 55]. However, during human corticogenesis, there are three types of cortical RG cells: ventricular zone (VZ) fRG cells, which give rise to VZ truncated radial glia (tRG) and outer radial glia (oRG, also known as basal RG) cells, located in the outer subventricular zone (outer SVZ, OSVZ) [55–66]. Prior to cortical neurogenesis, cortical neuroepithelial cells divide symmetrically to amplify the founder pool. Once neurogenesis starts, these cells are believed to directly transform into cortical fRG cells, which then divide asymmetrically to self-renew and produce intermediate progenitor cells (IPCs) for PyNs (PyN-IPCs). Neurogenic fRG cells in the cortical VZ sequentially generate distinct subtypes of PyNs in an inside-out pattern, with deep-layer PyNs born first, followed by PyNs of upper layers (Fig. 2a, b) [50–52, 67–69].

Cell lineage analysis shows that at the end of cortical neurogenesis, mouse cortical fRG cells progress from generating PyNs to astrocytes, oligodendrocytes, and a subpopulation of GABAergic olfactory bulb interneurons (OBINs) [48–51, 70–79]. However, there has been a fundamental gap in understanding how these diverse cell subtypes are generated. Our recent studies using genetic labeling methods in mice have demonstrated that during the cortical gliogenesis phase, fRG cells continue to divide asymmetrically to self-renew and produce a different type of IPC that is multipotent (tri-potential), which we have termed basal multipotent IPCs (bMIPCs) [80]. These bMIPCs undergo several rounds of mitosis and generate cortical astrocyte lineage-restricted IPCs (APCs), oligodendrocyte lineage-restricted IPCs (OPCs), and cortex-derived OBIN lineage-restricted IPCs (OBIN-IPCs) (Fig. 2a). These lineage-restricted IPCs then divide symmetrically to generate cortical astrocytes, oligodendrocytes and OBINs, respectively [80, 81].

Further research has shown that the molecular features of this process are consistent with known transcriptional mechanisms for generating oligodendrocytes, astrocytes, and OBINs. Cortical fRG cells generate bMIPCs in the SVZ that express epidermal growth factor receptor (EGFR), ASCL1, and OLIG2/1. When bMIPCs give rise to cortical APCs, they maintain the expression of EGFR and OLIG2/1 but downregulate ASCL1 expression, whereas OPCs maintain ASCL1 and OLIG2/1 expression but downregulate EGFR. Conversely, OBIN-IPCs maintain ASCL1 and EGFR expression but downregulate OLIG2/1 expression (Fig. 2a) [80–82].

In 1983, *in vitro,* studies demonstrated the existence of bi-potent glial-IPCs (O-2A cells) that give rise to both oligodendrocytes and astrocytes [83]. This study was not confirmed *in vivo* for a long time [84], but it is now accepted that both cortical OPCs and APCs are derived from the same population of IPCs, identified by us and others in the mouse and human cortex [60, 80, 85–91]. Furthermore, our genetic labeling experiments show that cortex-derived OBINs are also generated from these IPCs (bMIPCs) during development (Fig. 2a) [80, 85].

Cortical neurogenesis and gliogenesis always first occur in the rostroventral cortex, followed by the lateral, dorsal, and caudomedial cortex (Fig. 2c) [80, 92]. This is regulated by gradients of FGF, WNT-BMP, SHH signaling, and their contribution to the gradient expression of cortical patterning transcription factors (*Nr2f1, Pax6, Sp8, Lhx2*, and *Emx2*) in the cerebral cortex [4, 5, 22, 31–34, 93–97]. For example, neurogenesis occurs in the mouse ventrolateral cortex from E10.5 to E16.5, followed by gliogenesis, whereas neurogenesis in the medial cortex may occur from E12.5 to E18.5, after which gliogenesis begins.

In the human cerebral cortex, neuroepithelial cells start to convert into fRG cells around gestational week 7 (GW7) [59, 98, 99]. Then, fRG cells undergo asymmetric cell division to self-renew and produce PyN-IPCs around GW8, which may exclusively differentiate into deep cortical layer PyNs [56, 59, 60, 98, 100]. After approximately 9 weeks of neurogenesis (around GW16), human cortical fRG cells give rise to oRG and tRG cells [59, 60, 62]. oRG cells inherit the long basal fibers of fRG cells, while tRG cells inherit the apical domains of fRG cells [59, 60, 62, 101]. Both oRG and tRG cells can self-renew. The emergence of the cortical OSVZ in higher-order mammals is fundamentally different from that in mice [64]. oRG cells in the OSVZ mainly generate upper layer cortical PyNs [56, 57, 59, 61, 98, 102–104], and this process in humans lasts around 10 weeks from GW17 to GW26 (Fig. 2d, e) [55, 60].

In contrast to neurogenic oRG cells, our studies suggest that tRG cells in the cortical VZ primarily generate cortical glia [54, 55, 60]. After tRG cells are generated from fRG cells, a subpopulation of putative primed/active tRG cells starts to express EGFR and ASCL1. Initially, this population of primed tRG cells generates PyN-IPCs, but soon they undergo a neurogenesis-to-gliogenesis switch and generate bMIPCs that express ASCL1, EGFR, and OLIG1/2 [55, 60]. These bMIPCs then give rise to cortical APCs, OPCs, and OBIN-IPCs (Fig. 2d) [60]. Thus, this process in the human cortex is similar to that in the mouse cortex [60, 80], suggesting that mammalian cortical gliogenesis is evolutionarily conserved and that the presence of EGFR-expressing progenitors in the cortical VZ/SVZ is a strong signal for the onset of cortical gliogenesis in both mice and humans [55, 60, 80, 87].

As mouse cortex development proceeds after birth, fRG cells gradually give rise to human tRG-like NSCs, also known as B1 NSCs, which mainly generate OBIN-IPCs [70, 76, 105–108]. During aging, the number of NSCs and neurogenesis in the SVZ of the lateral ventricle declines [109], but the process of OBIN genesis persists throughout life in mice [106, 109]. On the other hand, while human postnatal cortical tRG cells (B1 NSCs) also generate OBIN-IPCs, this process only occurs during the first year of life. In the adult human SVZ, very few newly born OBINs are detected [110–118].

I propose that in both mouse and human brains, PyN genesis and gliogenesis first occur in the rostroventrolateral cortex, followed by the dorsal and caudomedial cortex [60, 80, 92]; this is a consistent rule in mammalian cortical development. For example, fRG cells in the human ventral cortex might start to generate PyNs at GW8, whereas fRG cells in the medial cortex might continue to expand their population by symmetric division and might not produce PyNs until GW10. At GW16, fRG cells in the ventral cortex give rise to oRG and tRG cells, whereas fRG cells in the medial cortex start to do so around GW18 [60]. Indeed, at GW18, we observe many human cortical APCs (GFAP^+^ HOPX^+^ cells) in the ventral cortex, whereas very few APCs are seen in the dorsal and medial cortex [60]. It is believed that the length of the human cortical neurogenic period is more than 130 days (GW8–GW26); however, a single fRG cell and its progeny oRG cell may generate cortical PyNs for only 110 days [55, 58, 98, 119, 120].

## “Slow and Multistep” Processes Fundamentally Underlie Cortical Neurogenesis during Evolution

Asymmetric division of stem cells is the key mechanism to maintain the balance between self-renewal and differentiation. During the cortical neurogenic stage, RG cells undergo asymmetric divisions into one daughter cell equivalent to the RG cell itself and another daughter cell committed to becoming a PyN-IPC. In the developing mouse cortex, a single neurogenic fRG cell divides 5-6 times and produces 8–9 PyNs that are distributed in both deep and upper layers (Fig. 2a, b) [48]. Mouse cortical neurogenic fRG cell cycle length ranges from 18–26 hours [120–123]. Compared with other mammals, mouse cortical fRG cells have a short cell cycle and short neurogenic period. Mice may also have the shortest length of gestation among mammals [120]. As noted above, individual mouse cortical fRG cells only have 6 days to generate PyN-IPCs and then switch to cortical gliogenesis (Fig. 2a) [48, 49, 80, 85, 91], resulting in mouse cortical fRG cells not undergoing strict self-renewal because their potential progressively changes and they generate different progenies over the course of cortical neurogenesis (Fig. 2a, b) [48–51, 75, 124].

In contrast to mice, non-human primate and human cortical RG cell cycles can be up to 44-63 hours, and the lengthening of cortical RG cell cycles is a well-documented feature of primates with larger brains [66, 120, 125–127]. Human cortical RG cells likely have the longest cell cycle and the longest neurogenic period. Humans also have the longest gestation period among primates [119, 120, 128]. Longer cell cycles are believed to maintain genomic fidelity, reduce the rate of somatic mutation, regulate cell fate, and ensure stem cell lineage fidelity [129, 130]. Therefore, human cortical RG cells undergo much stricter self-renewal than mice (Fig. 2d, e). I propose that the lengthening of the cortical RG cell cycle is an essential prerequisite for increasing the RG neurogenic period, whereas shortening the RG cell cycle results in shortening the neurogenic period and initiating cortical gliogenesis. Longer cell cycles and longer neurogenic periods of cortical RG cells inevitably promote a “slow and multistep” manner of neurogenesis in the human cortex. For example, a single neurogenic human cortical fRG cell in the lateral cortex may divide approximately 30 times (from GW8 to GW16) and generate PyN-IPCs, which may produce 30-45 PyNs distributed in deep layers. Around GW16, fRG cells give rise to tRG and oRG cells. A single human oRG cell in the lateral OSVZ may also divide 30 times (from GW16 to GW24) and generate PyN-IPCs, which may produce 30-45 PyNs distributed in upper layers (Fig. 2d, e). In the mouse cortex, a single cortical fRG cell may produce only 2 PyNs in layer 6, whereas a single human cortical fRG cell may produce more than 20 PyNs in layer 6, which exhibit similar morphological, physiological, molecular, and connectional features (Fig. 2d, e), and then they start to generate PyNs in layer 5. The acquisition of a “slow and multistep” manner of cortical neurogenesis is therefore a fundamental property that leads to the evolution of the human cortex.

What are the molecular mechanisms underlying the lengthening of cell cycles and the neurogenic period of human cortical RG cells? We and others have looked for fundamental molecular mechanisms that regulate both mouse and human cortical development and neurogenesis (Fig. 2f, g). For example, cortical neurogenesis is protected by GLI3R-mediated suppression from the ventralizing effects of SHH and FGF signaling [35–42]. *Pax6*, *Dmarta2*, *Emx2*, and *Lhx2* expression in the cortical RG cells promote and protect neurogenesis [22, 31–34, 131]. NOTCH signaling regulates cortical RG cell quiescence and self-renewal [56, 132–135]. FGF-ERK signaling promotes cortical RG cell proliferation and self-renewal [12, 54, 93, 136–141]. SHH signaling, especially in mice, may promote cortical neurogenic RG cell proliferation [55, 142–144] (Fig. 2f).

While the transcriptional profiles of human and mouse neocortical RG cells are broadly conserved during neurogenesis, studies have also identified some signaling pathways that are much stronger in human cortical RG cells. Among these signaling pathways, FGF-ERK, BMP7-SMAD, LIF/LIFR, GLI3R, and PDGFD–PDGFRB signaling are stronger in humans than in mice (Fig. 2g) [54, 55, 58, 145–147]. FGF-ERK signaling is required for normal RG cell proliferation and self-renewal. On the other hand, BMP7-SMAD, LIF/LIFR, GLI3R, and PDGFD–PDGFRB [148] signaling pathways play crucial roles in maintaining human cortical RG cell self-renewal and quiescence. Moreover, BMP7-SMAD signaling inhibits cortical gliogenesis, which may greatly increase the human cortical neurogenic period [54, 55].

**Fig. 3:**
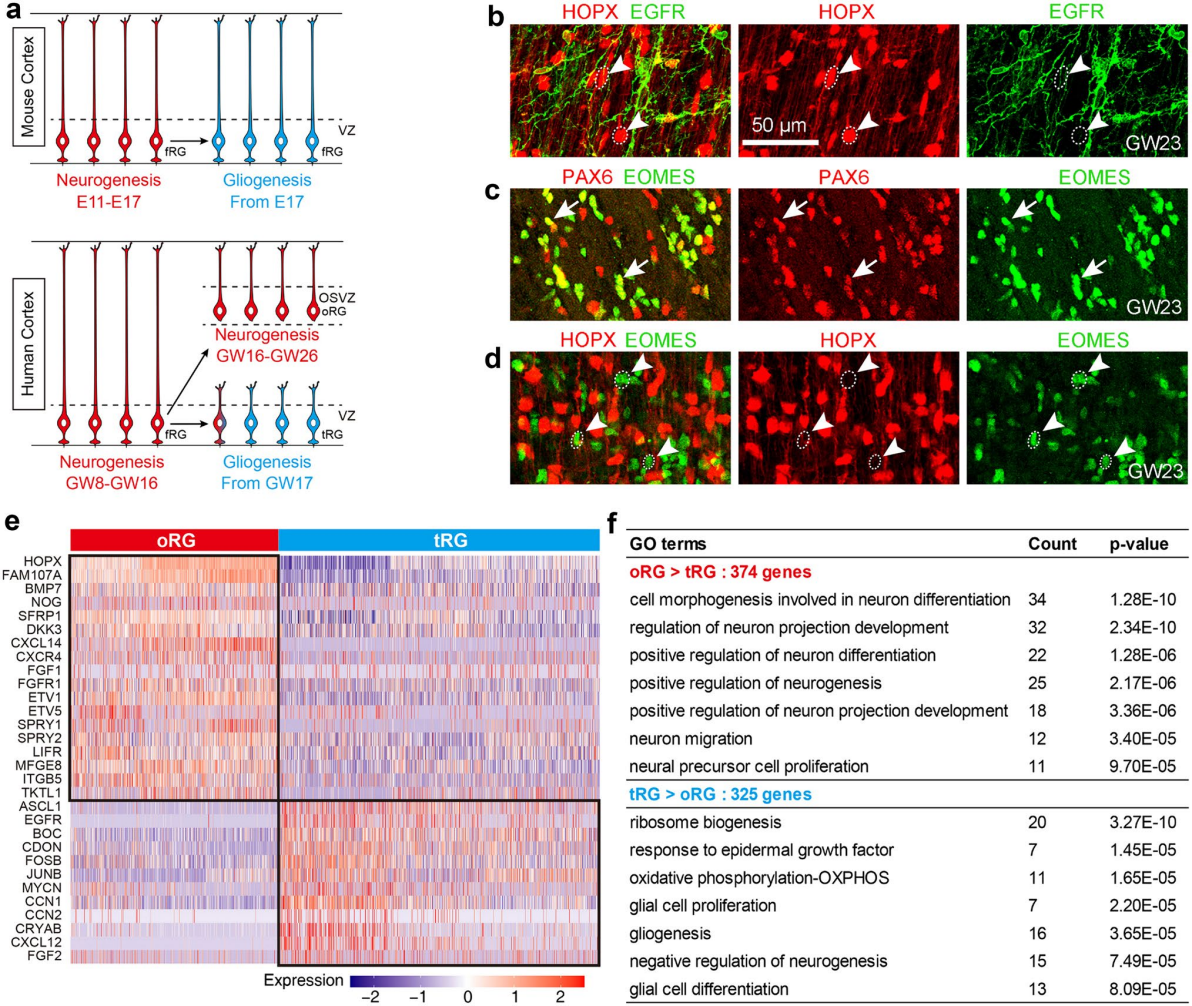
Human cortical oRG cells and tRG cells exhibit distinct transcriptional signatures. **a** Schematic summarizing the process of mouse and human cortical RG neurogenesis and gliogenesis. **b** Immunohistochemical analysis shows that human oRG cells have a larger soma (more than 10 µm in diameter, arrowheads) and do not express EGFR. Note that HOPX^+^ EGFR^+^ cells are cortical APCs, as they have smaller soma size. **c** PAX6^+^ oRG cells produce TBR2 (EOMES)^+^ PyN IPCs that maintain PAX6 expression (arrows). **d** Very strong HOPX-expressing oRG cells generate TBR2 (EOMES)^+^ PyN IPCs (arrowheads) that never express HOPX, suggesting that oRG cells have longer cell cycles. **e** scRNA-Seq analysis of molecular profiles of human cortical RG cells at GW22, GW23, and GW26. Heat map of selected differentially expressed genes for oRG cells versus tRG cells. **f** Selected GO terms supporting that oRG cells are neurogenic while tRG cells are gliogenic. Images (**a**, **e**, **f**) are reprinted with permission from Li *et al.*, (2024) [55]. Images (**b**–**d**) are adapted from Yang *et al.*, 2022 [60].

As mentioned above, oRG cells in the OSVZ mainly generate upper cortical layer PyNs [56, 57, 59, 61, 98, 102–104], and this process in humans lasts around 7-10 weeks from GW16 to GW26 (Fig. 2d–g) [55, 60]. This conclusion has been supported and expanded upon by the analysis of the transcriptome of human oRG and tRG cells using single-cell RNA sequencing (scRNA-Seq) (Fig. 3) [55, 58]. To define the major transcriptional features of gliogenic tRG and neurogenic oRG cells, we performed differential gene expression analysis between neurogenic oRG cells and gliogenic tRG cells in GW22, GW23, and GW26 cortex [88]. Although both human cortical oRG and tRG cells are derived from fRG cells, they exhibit distinct transcriptional signatures. tRG cells have higher CXCL12/CXCR4, SHH, and EGFR signaling, which promotes cortical gliogenesis (Fig. 3a, e, f) [85, 149–153]. On the other hand, higher BMP7, GLI3R, CXCL14/CXCR4, and FGF-ERK signaling in oRG cells promote self-renewal and quiescence and protect neurogenesis (Fig. 3e) [28, 54, 55, 142]. Notably, strong expression of BMP7-SMAD signaling, and its downstream target CXCL14, are absolutely required for maintaining stem cell identity [154, 155]. CXCL14/CXCR4 signaling may also block CXCL12/CXCR4 signaling [156].

Neurogenesis in human cortical oRG cells is further slowed down. Our immunohistochemical analyses found that a subpopulation of human cortical tRG cells express EGFR, whereas oRG cells do not (Fig. 3b) [60]. scRNA-Seq analysis of fluorescence-activated cell sorting EGFR^+^ cells isolated from the frontal lobe of the human cerebral cortex between GW21 and GW26 supported this conclusion [60, 87]. We also observed that strong HOPX-expressing oRG cells with large soma size (more than 10 µm in diameter) generate PyN-IPCs that express EOMES (TBR2) but never express HOPX (Fig. 3c, d) [60], further suggesting that oRG cells have a longer cell cycle and undergo stricter self-renewal. EOMES-expressing PyN-IPCs also express PPP1R17, which regulates G1/S transition and slows PyN-IPC cell cycle progression [119]. All these observations reveal that oRG neurogenesis follows a typical “slow and multistep” manner.

*In vitro* studies have shown that while enhancing BMP signaling alone promotes terminal astrocytic differentiation, exposure to both BMP and FGF2 (ERK signaling) maintains the stem cell character of cultured progenitor cells [157, 158]. We speculate that higher FGF-ERK activity in oRGs antagonizes quiescent BMP7 signaling through phosphorylation of the SMAD linker domain [159, 160]. oRG cells also express higher levels of NOG, a secreted BMP antagonist [55, 58]. Therefore, the intricate balance of quiescence, proliferation, self-renewal, and differentiation (neurogenesis) is well controlled by those signaling pathways that are at higher levels in human cortical oRG cells, resulting in oRG cells playing a key role in the increased generation of PyNs that constitutes the foundation of neocortex expansion. At the end of cortical neurogenesis, oRG cells may directly transform into astrocytes, but not generate OPCs [60], because BMP signaling is further enhanced in the cortex from the second trimester to the third trimester [55].

## ERK Signaling Drives the Evolutionary Expansion of the Mammalian Cerebral Cortex

The human cerebral cortex has the largest number of neurons (16.3 billion) among primates (Fig. 1). Two main factors determine cortical PyN numbers and the surface area of the cerebral cortex. One is the size of the cortical neuroepithelial and fRG cell pool present in the VZ at the beginning of cortical neurogenesis, which largely determines the PyN number. This is known as “the radial unit hypothesis” originally proposed by Rakic [1, 161–163]. The second is the length of the cortical neurogenic period [120, 126, 164]. As indicated above, humans have the longest cortical neurogenic period [55, 59, 60, 119, 120], allowing cortical RG cells to produce more PyNs. According to the “radial unit hypothesis” model, an increase in the neurogenic period appears to contribute a linear increase in the number of cortical PyNs within each radial cortical column (Fig. 2b, e), which also contributes to an increase in cortical thickness [165–168].

Remarkable progress has been made over the past thirty years in identifying potential mechanisms that contribute to mammalian cortical development, expansion, and evolution [1, 14, 46, 50–52, 68, 98, 102, 120, 126, 146, 161, 163, 169–175]. However, unifying molecular principles, if they exist, have not yet been identified that have driven progressively larger cortices during mammalian evolution. As indicated in Fig. 1, I believe that there must be a common rule that drives mammals to generate more and more cortical PyNs during evolution, which contributes to an expansion of the cortex and an increase in brain volume across primates [176–178]. I postulate, that we may have recently identified such a core mechanism that drives mammalian cortical expansion during evolution [54].

By comparing mouse, ferret, monkey, and human developing cortex, we found that *BMP7* is expressed by increasing numbers of cortical RG cells [55]. We demonstrated that BMP7-SMAD signaling represses EGFR expression in cortical RG cells and inhibits SHH signaling by promoting GLI3R formation [54, 55]. Therefore, BMP7 in cortical RG cells promotes neurogenesis, inhibits gliogenesis, and thereby extends the neurogenic period, resulting in the production of a large number of cortical PyNs. As mentioned above, BMP signaling also functions to maintain stem cells in a quiescent state and sustain stem cell self-renewal [28, 154, 155, 179–182].

**Fig. 4:**
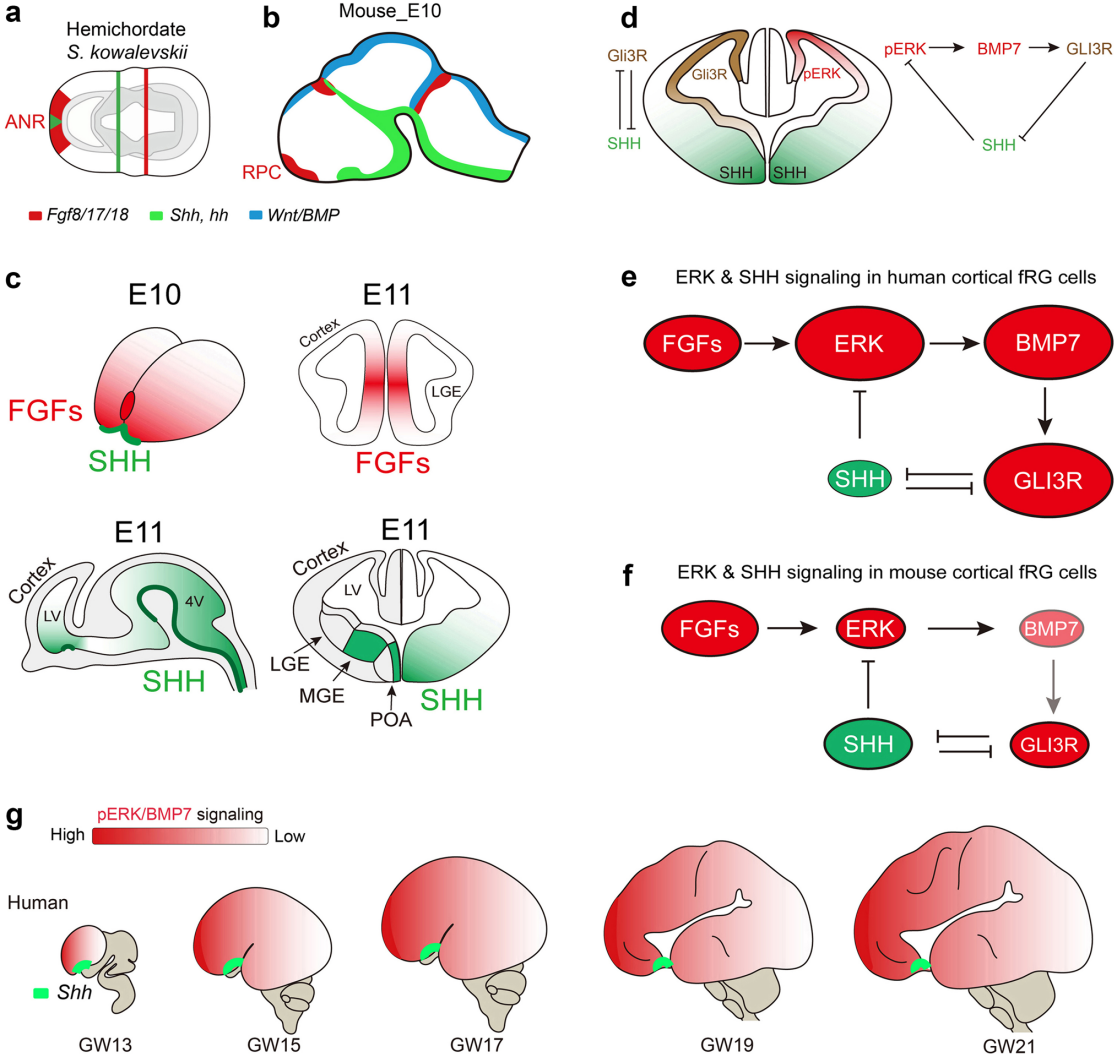
ERK signaling drives the evolutionary expansion of the mammalian cerebral cortex. **a**, **b** The ANR expressed FGFs in hemichordates (500 million years ago) and mice. **c** During mouse early forebrain development, FGFs are expressed in the rostral patterning center, whereas SHH is expressed in POA stem/progenitor cells and MGE-derived neurons. **d**, **e** At the beginning of cortical neurogenesis in the most recent ancestor to all mammals, it is assumed that there was already a subset of cortical fRG cells that expressed relatively higher levels of pERK. Elevated ERK signaling in these cortical fRG cells promotes BMP7 expression, which increases GLI3R generation and represses SHH signaling. A decrease in SHH signaling in cortical fRG cells further enhances ERK signaling. Therefore, the ERK-BMP7-GLI3R-SHH signaling pathway in cortical fRG cells participates in a positive feedback loop, which expands the cortical fRG cell pool, increases the length of the cortical neurogenic period, and thus can drive increases in cortical surface area and neurogenesis; this may underlie these features characteristically observed in evolutionarily more advanced mammals. **f** SHH signaling underlies cortical evolutionary dwarfism in mice. During mouse cortical development and evolution, smaller brains with smaller lateral ventricle volumes may result in higher SHH concentration in the cerebral spinal fluid and thus can increase SHH signaling in the cortex, which antagonizes ERK signaling. Relatively weak ERK signaling fails to induce *Bmp7* expression in the dorsal cortical RG cells, resulting in a shortened period of cortical neurogenesis (for example, from more than 130 days in humans to about 7 days in mice). **g** ERK signaling is elevated in human cortical fRG and oRG cells during development and evolution and induces *BMP7* expression that antagonizes SHH signaling. Images are reprinted with permission from Sun *et al.*, 2024 [54].

What signals induce *BMP7* expression in cortical RG cells during development and evolution? It turns out that FGF-ERK signaling is crucial. FGF signaling is required for neural induction, which begins during early gastrulation. The anterior neural ridge (ANR) is located in the anterior neural plate of vertebrates and expresses FGFs (*FGF8/17/18*), which are important for patterning the telencephalon [183–185]. This conserved mechanism dates back at least 500 million years, as the ANR-like genetic regulatory program of FGF signaling is present in hemichordates and amphioxus (Fig. 4a) [186]. The ANR-derived rostral telencephalic patterning center (RPC) produces FGFs (Fig. 4b, c) [7–9, 184, 187]. FGFs and their downstream targets, *Spry1* and *Spry2,* are mainly expressed in the presumptive septum and their expression extends into the mouse medial cortex during early cortical neurogenesis [7, 141, 187]. Interestingly, *Bmp7* is also expressed in the medial cortex [55, 188]. Moreover, previous studies have shown co-expression of *Fgf8* and *Bmp7* in the mouse ANR [9]. These observations indicate that *Bmp7* expression might be induced by FGF signaling. Indeed, when we conditionally overexpressed *Fgf8* in the cortex using *hGFAP-Cre* transgene lines, strong *Bmp7* and HOPX expression was observed in the E14.5 neocortical VZ [54].

**Fig. 5:**
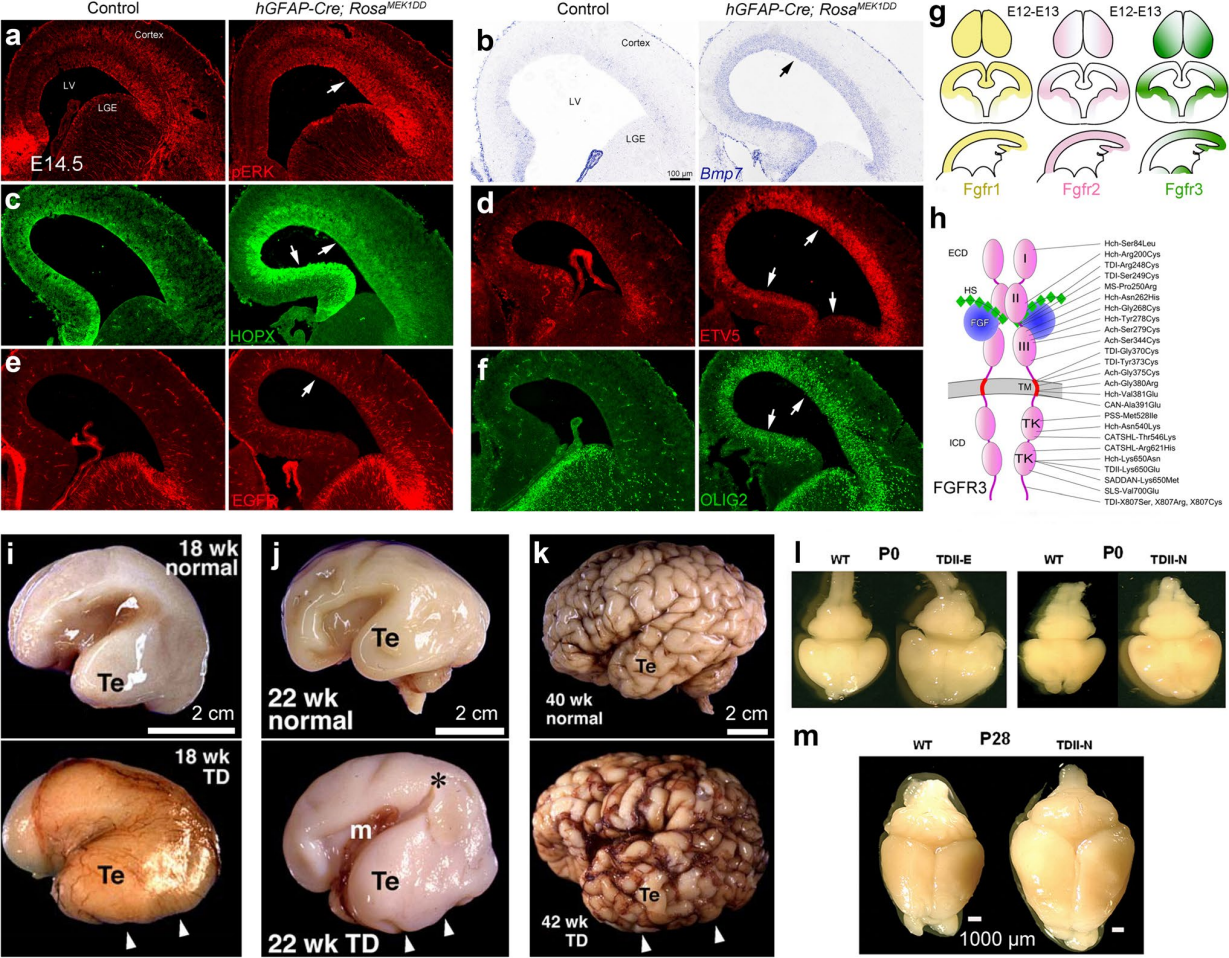
Constitutive ERK signaling induces *Bmp7* expression in cortical RG cells and can drive cortical expansion. **a**–**f** Expression of pERK (phosphorylated ERK), BMP7, HOPX, ETV5, EGFR, and OLIG2 is increased in the cortex of *hGFAP-Cre; Rosa*^*MEK1DD*^ mice at E14.5 (arrows); reprinted with permission from Sun *et al.*, 2024 [54]. **g** Expression patterns of *Fgfr1*, *Fgfr2*, and *Fgfr3* in E12-E13 mouse cerebral cortex; reprinted with permission from Iwata and Hevner (2009) [190]. Note the low rostral-high caudal *Fgfr3* expression gradient. **h** The mutational spectrum of human *FGFR3*. Gain of function *FGFR3* mutations result in dwarfism syndromes, ranging from achondroplasia and hypochondroplasia to the more severe neonatal lethal thanatophoric dysplasias TDI and TDII; reprinted with permission from Ornitz and Legeai-Mallet (2015) [204]. **i**–**k** Temporal lobe enlargement and abnormal sulci in GW18, GW22, and GW40 TD fetuses; reprinted with permission from Hevner (2005) [205]. **i**, **m** Brain morphology of TD mouse at P0 and P28. Note that TD mice have an enlarged cerebral cortex; reprinted with permission from Lin *et al.*, (2003) [211].

The interaction of FGFs with FGFRs results in phosphorylation and nuclear translocation of ERK1/2, which phosphorylates target transcription factors [139, 189–193]. ERK also directly regulates gene expression via its DNA binding activity [194–196]. ERK signaling can be activated in the *Rosa*^*MEK1DD*^ mouse [151, 197, 198], which contains a Cre-inducible constitutively active rat *Map2k1* allele. We introduced the *hGFAP-Cre* allele into the *Rosa*^*MEK1DD*^ mice and observed that strong activated ERK induced *Bmp7* expression in the E14.5 cortical VZ (Fig. 5a–f). Furthermore, HOPX, ETV5, EGFR, and OLIG2 expression were also strongly upregulated (Fig. 5a-f). We have also shown that loss of cortical ERK signaling results in loss of *Bmp7* and HOPX expression [54], premature neural differentiation, and depletion of cortical RG cells [54, 136–138]. Therefore, ERK signaling is required for cortical expression of *Bmp7* and HOPX and maintaining self-renewal and the undifferentiated state of cortical RG cells. Notably, abnormal sustained FGF-ERK activity also induces a switch from cortical neurogenesis to gliogenesis [54, 140, 151, 199].

In the E11 mouse cortex, FGF-ERK signaling exhibits a rostral-high to caudal-low gradient, whereas SHH signaling exhibits a ventral-high to dorsal-low and caudal-high to rostral-low gradient, which is opposite (Fig. 4b–d) [2, 4, 5, 200]. Gain and loss of function studies demonstrated that SHH-SMO signaling antagonizes ERK-BMP7 signaling in mouse cortical fRG cells and sustained SHH-SMO activity also promotes the onset of cortical gliogenesis [54, 55, 85, 152, 201], suggesting that SHH signaling contributes to the shortening of the mouse cortical neurogenic period.

By integrating analyses of published mouse, ferret, monkey, and human cortical scRNA-Seq datasets with immunostaining and mRNA in situ hybridization experiments, we have provided strong evidence that ERK signaling is progressively higher in cortical RG cells in evolutionarily more advanced species [54]. We propose that similar to the mouse cortex, cortical RG cell expression of *BMP7* in other mammals, including humans, is also induced by ERK signaling, consistent with *BMP7* expression gradually upregulating in mouse, ferret, monkey, and human cortical RG cells during evolution [54, 55, 146, 147, 202]. Based on these findings, I believe that we have identified a core mechanism that drives cortical expansion and evolution.

We propose that ERK signaling drives the evolutionary expansion of the mammalian cerebral cortex (Fig. 4d–g) [54]. The most recent ancestor to all mammals, approximately 100 million years ago, is assumed to have already been somewhat gyrencephalic (Fig. 1) [120, 203], and is assumed to have had a subset of fRG cells in the developing dorsal cortex that expressed relatively higher levels of ERK. We showed that elevated ERK signaling in cortical fRG cells induces *BMP7* expression, which then promotes GLI3R production and represses SHH signaling [54, 55]. A decrease in SHH signaling in cortical fRG cells further enhances ERK signaling. Therefore, ERK-BMP7-GLI3R-SHH signaling in cortical fRG cells participates in a positive feedback loop (Fig. 4d, e), which increases the size of the cortical RG cell pool, restrains but allows neurogenesis while inhibiting gliogenesis, and thus increases the length of the neurogenic period. We hypothesize that SHH concentration in cerebrospinal fluid is reduced in the enlarged lateral ventricle of big-brained mammals during cortical development and evolution; this could contribute to a further decrease in SHH signaling and an increase in ERK-BMP7-GLI3R signaling in cortical fRG cells (Fig. 4g).

Similarly, ERK-BMP7-GLI3R expression in oRG cells in the cortical OSVZ continues to participate in a positive feedback loop because the primary cilia of oRG cells do not contact cerebrospinal fluid in the lateral ventricle and thus may not receive SHH signaling. Indeed, scRNA-Seq analysis of the human cortex at GW18, 22, 23, and 26 [88], never reveals SHH target gene GLI1 expression in human oRG cells, whereas GLI1 expression is detected in tRG cells. SHH-SMO signaling promotes EGFR expression, whereas BMP7 and GLI3R repress EGFR expression in cortical RG cells [54, 55]. This is why tRG cells express EGFR, but oRG cells do not [55, 60, 87]. As mentioned above, EGFR-expressing primed/active tRG cells in the cortical VZ are mainly gliogenic, while oRG cells in the cortical OSVZ continue to generate PyNs. This two-germinal-zone system in the human developing cortex ensures that the onset of tRG gliogenesis occurs in the VZ, while oRGs in the OSVZ produce PyNs [54, 55, 60], the latter increases the length of the neurogenic period enabling the generation of more PyNs [120, 126, 164].

The lissencephalic mouse is believed to have originated from a larger and gyrencephalic ancestor (Fig. 1) [120, 203]. Based on our model, I propose that SHH signaling drives the phyletic dwarfism of the mouse cortex. Smaller brains with smaller lateral ventricles may result in higher concentrations of SHH that bathe the cortical VZ and antagonize ERK signaling during development and evolution (Fig. 4d, f), resulting in relatively lower ERK-BMP7 signaling in mouse cortical fRG cells [54], which reduces the size of the cortical RG cell pool and shortens the neurogenic period. I think that this is the major reason that the mouse cortex only has ~13.7 million neurons, whereas the human cortex has ~16.3 billion neurons (Fig. 1) [43–45].

Our proposed principle of cortical development, expansion, and evolution is supported by multiple types of genetic evidence. The normal development and expansion of the human cortex provide strong evidence that ERK signaling drives the evolutionary expansion of the mammalian cortex. The rostral patterning center releases FGFs, and promotes the size of the rostral cortex and basal ganglia [2, 4, 7, 8, 10–12, 14]. FGF-ERK-BMP7 signaling is elevated during cortical development and evolution, playing a crucial role in expanding the size of the RG cell pool and increasing the length of the neurogenic period [54, 55], resulting in a significant expansion of the human frontal cortex, the most highly developed and expanded brain structure in mammal evolution [1, 102].

Probably the strongest genetic evidence that supports our principle is the development and expansion of the human temporal and occipital cortex in Thanatophoric Dysplasia (TD) patients (Fig. 5g–k). TD is a lethal form of short-limbed dwarfism caused by mutations of *FGFR3*, which lead to constitutive activation of FGFR3 tyrosine kinase activity [190, 204, 205]. *FGFR3* is expressed in a high caudomedial-low rostrolateral gradient in the human [206], ferret [207], and mouse cortical neuroepithelial and RG cells (Fig. 5g, h) [11, 208]. *FGFR3* gain of function mutation in TD patients significantly strengthens ERK signaling [209, 210], which may further enhance *BMP7* expression, causing the significant expansion of the temporal and occipital lobes in TD patients [190, 205]. Notably, the TD mouse provides an excellent model for the human TD, as they also exhibit the expansion of the cortex (Fig. 5l, m) [204, 209, 211–215], further providing evidence that FGF-ERK signaling can drive cortical expansion during evolution.

Finally, the case report that heterozygous loss of function mutations in the human *BMP7* gene cause small head size, provides additional genetic evidence for BMP7’s function in promoting cortex size [216].

## SHH Signaling is Not a Key Regulator of Primate Cortical Expansion and Folding

**Fig. 6:**
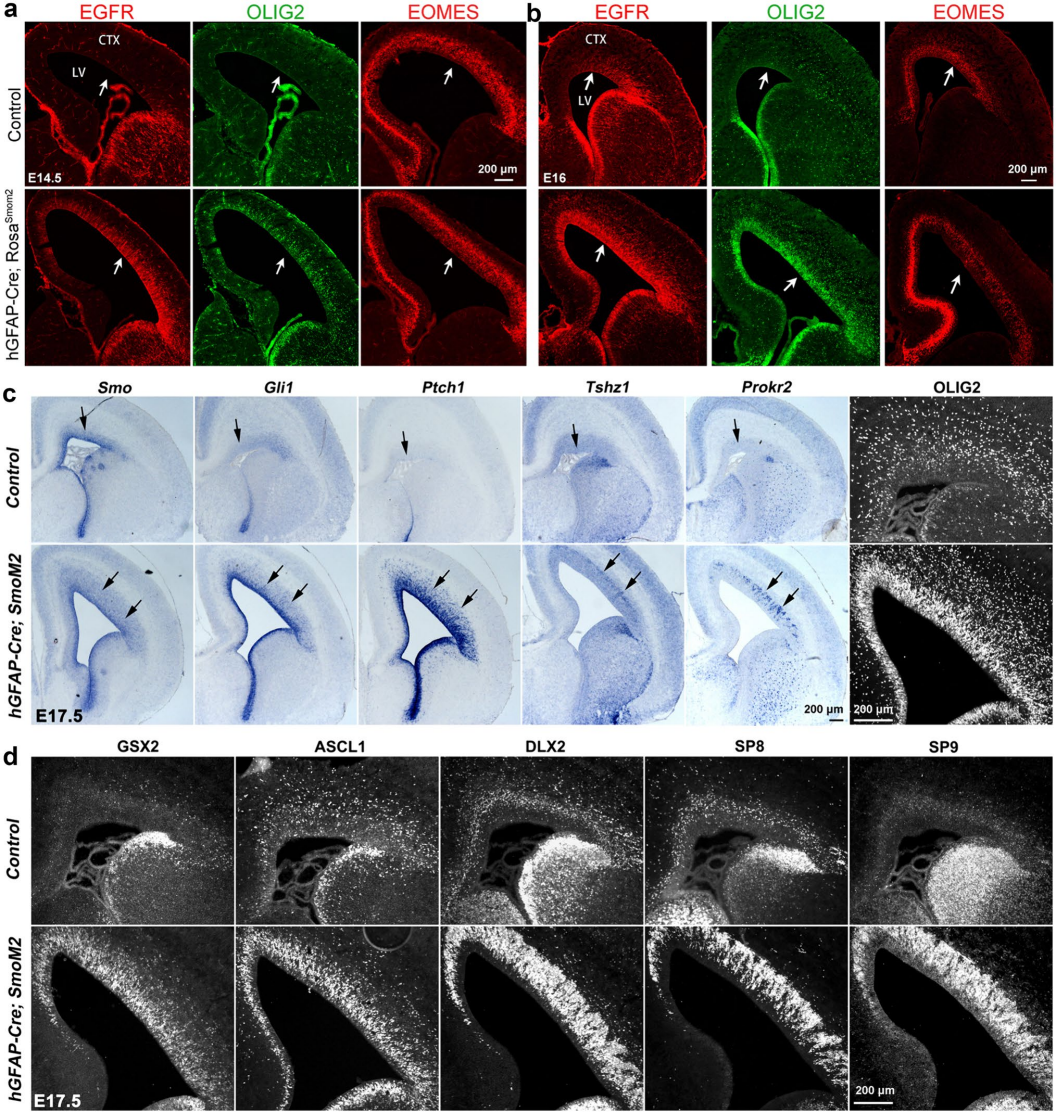
Constitutive SHH-SMO signaling promotes cortical OBIN genesis. **a**–**d** Higher levels of SHH-SMO signaling promote cortical RG cell and IPC proliferation, induce EGFR and OLIG2 expression, expand the cortical VZ/SVZ, and strongly induce GSX2 and DLX2 expression, favoring the generation of OBINs. Images are reprinted with permission from Sun *et al.*, (2024) [54], Li *et al.*, (2024) [55], and Zhang *et al.*, (2020) [85].

Based on evidence that SHH-SMO signaling antagonizes ERK-BMP7 signaling in mammalian cortical RG cells [54, 55], I propose that SHH signaling drives cortical evolutionary dwarfism, exemplified by the lissencephalic mouse that originated from a larger and gyrencephalic ancestor [120, 203]. However, several studies have concluded, contrary to my proposal, that SHH signaling promotes cortical expansion and gyration [217–230]. It is well known that SHH is a strong mitogen and promotes the proliferation of NSCs and IPCs in the brain [4, 85, 143, 152, 218, 231]. For example, SHH signaling promotes the extensive and prolonged symmetric division of granule cell precursors, which then differentiate into mature cerebellar granule cells in the cerebellum, the most numerous neurons of the brain [232]. Dysregulation of the SHH pathway results in SHH-subtype medulloblastoma, one of the most common malignant brain tumors in children [233]. In the wild-type mouse brain, the cortical VZ/SVZ expresses higher levels of *Gli1* during the first postnatal week than before birth, indicating an increase in SHH signaling in the postnatal cortical VZ/SVZ [234]. This coincides with an increase in cortical OBIN genesis in the cortical SVZ after birth. Higher levels of SHH signaling are necessary for a Type C cell (derived from B1 NSCs) to produce 16-32 OBINs [235–237]. Therefore, SHH’s function does not appear to be part of the ERK-BMP7-GLI3R pathway that promotes the expansion of cortical PyN. Indeed, when SHH-SMO signaling is constitutively active in the cortex of *hGFAP-Cre; Rosa*^*SmoM2*^ mice, although the proliferation of cortical fRG cells and the cortical VZ length are increased (Fig. 6a), a large number of EGFR^+^ OLIG2^+^ cells are also generated (these cells are not present in the cortex of control mice at E14.5) (Fig. 6a), indicative of a switch from cortical neurogenesis to gliogenesis. At E16.5, while more EOMES^+^ PyN-IPCs are present in the medial cortex, these cells are reduced in the lateral cortex as more EGFR^+^ OLIG2^+^ cells are generated in this region (Fig. 6b). At E17.5, cortical fRG cells precociously generate large numbers of OBIN-IPCs in the SVZ of *hGFAP-Cre; Rosa*^*SmoM2*^ mice (Fig. 6c, d) [54, 55, 85]. We also ectopically overexpressed non-cholesterol-modified SHH (*SHHN*) in the E13.5 cortical VZ using in utero electroporation, and examined the cortex at E18.5. Similar to *hGFAP-Cre; Rosa*^*SmoM2*^ mice, we observed a large number of cells expressing OBIN markers [85].

**Fig. 7:**
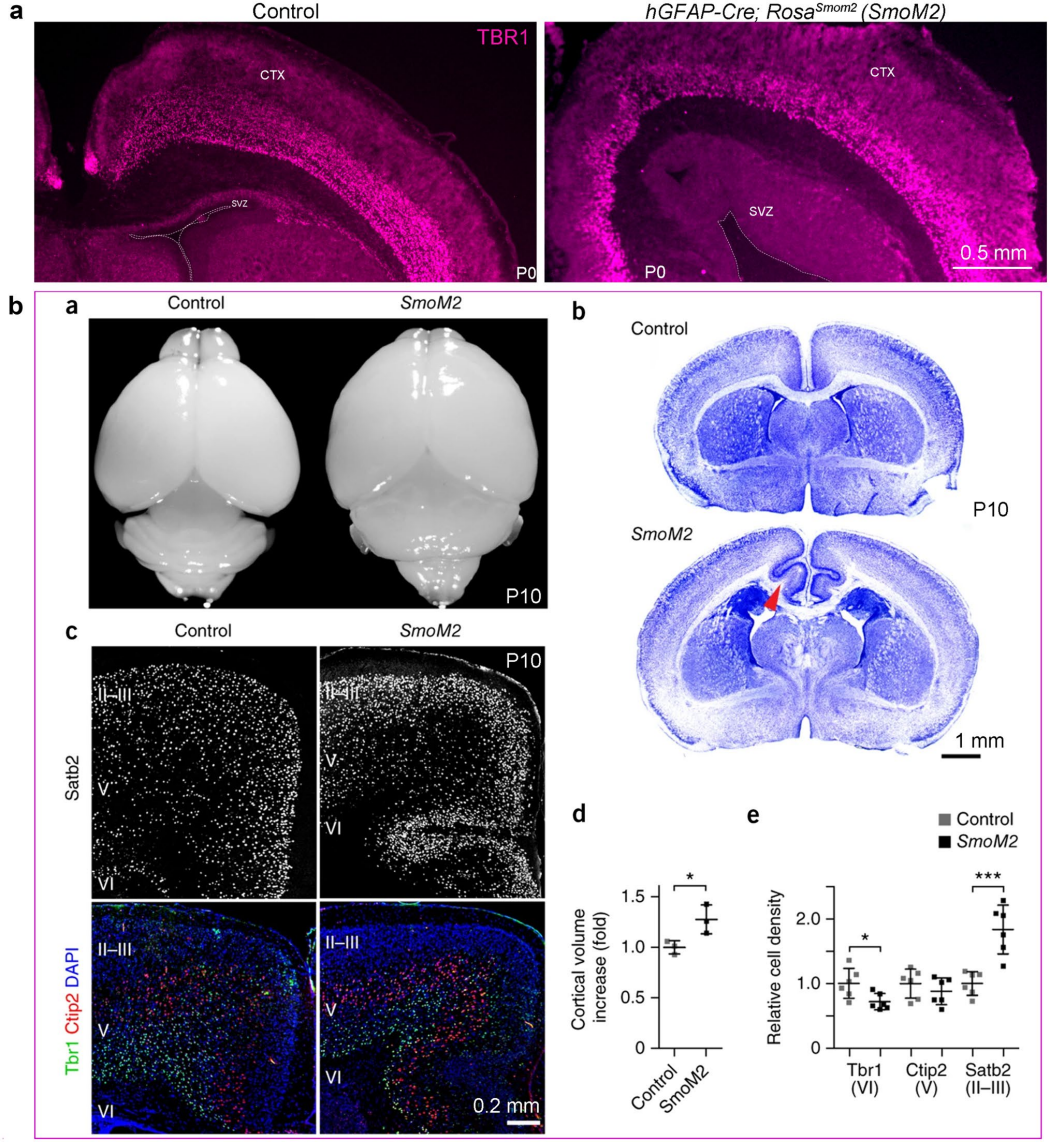
Constitutive SHH-SMO signaling promotes cortical abnormal folding. **a** Higher levels of SHH-SMO signaling reduce the number of TBR1^+^ PyNs in the deep cortical layers in *hGFAP-Cre; Rosa*^*SmoM2*^ mice at P0. Note that the thickness of the SVZ is expanded and the cortex is enlarged. **b** Higher levels of SHH-SMO signaling induce abnormal medial cortical folding and cortical expansion, reduce the number of TBR1^+^ PyNs in the deep cortical layers, and increase SATB2^+^ PyNs in the upper cortical layers at P10; reprinted with permission from Wang *et al.*, (2016) [218].

After birth, in *hGFAP-Cre; Rosa*^*SmoM2*^ mice, abnormal folding is observed in the medial cortex, the number of PyNs in the upper layer is increased, whereas the number of PyNs in the deep layer is reduced, and the thickness of the cortical SVZ is significantly expanded because OBIN-IPCs are robustly proliferating (Fig. 7a, b) [85, 218]. Therefore, higher levels of SHH signaling in the developing mouse cortex greatly disrupt the normal program of corticogenesis (Fig. 7a, b) [54, 55, 85, 201, 238]. This provides evidence against the idea that increasing SHH-signaling may underlie the evolutionary expansion of the primate cortex.

Finally, I have noted that abnormal cortical folding is observed in the medial cortex in transgenic mouse models used for studying cortical expansion, including *hGFAP-Cre; Rosa*^*SmoM2*^ [218], *Emx1-Cre; Gli3 *^*F/F*^ [239], *Emx1-Cre; Cep83 *^*F/F*^ [240], *Nes-Cre; Gpr161 *^*F/F*^ [226], and *hGFAP-Cre; Pik3ca*^*H1047R*^ (conditional activating mutations of *Pik3ca*) [241, 242] mouse lines. What are the molecular mechanisms underlying this folding phenotype? I suggest that because mouse medial cortical fRG cells express higher levels of ERK-BMP7-GLI3R signaling (similar to human cortical fRG and oRG cells) when they receive abnormal cell proliferation signals such as strong SHH signaling, they are able to sustain their self-renewal, maintain neurogenesis, and inhibit gliogenesis for a longer time than lateral cortical RG cells [54, 55], resulting in abnormal folding in the medial cortex.

## Human Cortical RG Cells Do Not Generate Cortical Interneurons

The mammalian neocortex contains two main neuronal subpopulations: cortical PyNs and cortical inhibitory GABAergic interneurons (CINs). In the mouse cortex, PyNs constitute about 80%, whereas CINs constitute 20% of all cortical neurons. Recent studies in mouse models and humans provide evidence that CIN dysfunction is associated with neurological and psychiatric diseases such as epilepsy, schizophrenia, bipolar disorders, and autism, highlighting their crucial roles in the development and organization of cortical networks that underlie a wide range of cortical and mental functions [243–251]. Studies over the past 30 years have demonstrated that nearly all mouse CINs originate from subcortical ganglionic eminences (GEs)-the medial and caudal ganglionic eminence (MGE and CGE) and preoptic area (POA) [251–266]. The lateral ganglionic eminence (LGE) may not generate CINs, as the dorsal LGE mainly generates OBINs and the ventral LGE mainly generates striatal projection medium spiny neurons [131, 258, 264, 267–281].

Although the LGE, MGE, and CGE of the human fetal brain have similar morphological features as mice [282], the origin of CINs in humans and non-human primates was controversial as previous studies reported that, in contrast to rodents, a large number of CINs in humans and non-human primates are generated from cortical progenitors [283–287]. In 2013, two papers published back-to-back revealed that similar to mice, most, if not all, human CINs are also derived from the subcortical GEs [110, 288]. They showed that markers of the MGE (NKX2-1 and LHX6), LGE (SP8 in the dorsal LGE and ISL1 in the ventral LGE), and CGE (NR2F2 and SP8) were very similar in the developing human, macaque monkey and mouse telencephalon. Monkey and human fetal brain slice cultures and BrdU labeling provided strong evidence that primate CINs are derived from GEs [110, 288]. These results show that the patterns of transcription factor expression and the tangential migration of CINs from the GEs to the cortex are conserved from rodents to primates during mammalian brain evolution [110, 288–290].

In 2019, a group of scientists in the field published a review paper [102] and commented on mammalian CIN generation: “experiments in rodents have demonstrated that inhibitory interneurons are produced in the GEs and tangentially migrate into the cortex, and to a large extent, the human fetal brain appears to share fundamental mechanisms for CIN development [290]. Landmark publications [110, 288] challenged the earlier notion that up to 65% of CINs may derive from the dorsal pallium [283] and demonstrated a predominantly ventral pallial origin for interneurons”. In 2021, two studies [291, 292], by conducting scRNA-Seq and in situ sequencing in developing human brains, further revealed that transcriptional programs that control interneuron specification, migration, and differentiation are evolutionarily conserved in mice and humans.

More recently, several papers have claimed the discovery of a cortical origin of some human CINs [293–298]. However, these results are inconsistent, and some are even contradictory. For example, previous studies showed that when dissociated cells from GW15.5 cortical OSVZ and VZ/SVZ were cultured with BrdU to label proliferating cells, after 7 days, BrdU^+^ GABA^+^ cells were not observed [56], suggesting that cortical progenitors do not produce inhibitory neurons around GW15-GW16. In contrast, lentiviral clone labeling of dissociated cortical cells from GW15 to GW18 indicated that individual human cortical progenitors generate both PyNs and CINs, and these cortex-derived interneurons were transcriptionally similar to CGE-derived CINs [293]. Another study using cell-type-resolved mosaicism revealed that a substantial portion of cortex-derived interneurons may contribute to parvalbumin^+^ CINs (MGE-like), whereas cortical NR2F2 (COUP-TFII)^+^ interneurons are most likely derived ventrally from the CGE [298].

In addition, some papers reported that human cortical neurogenesis ceases around GW19 [299], and reported that cortical oRG cells give rise to CGE-like CINs and/or cortical OPCs [86, 89, 295–297, 300]. Again, these results do not make sense as cortical oRG cells are mainly neurogenic and do not receive SHH signaling. In the mouse cortex, when SHH-SMO signaling is absent (*hGFAP-Cre; Smo*^*F/F*^), the presence of EGFR^+^ ASCL1^+^ OLIG2^+^ bMIPCs from cortical fRG cells is postponed, cortical gliogenesis is retarded, the cortical PyN genesis period is prolonged [54, 55], and cortical fRG cells fail to generate OBINs [85]. This is consistent with previous observations that SHH signaling is required for the generation of interneurons and oligodendrocytes in the ventral GEs [231, 301, 302]. In the mouse dorsal cortex, SHH signaling appears to favor the specification of OBINs but not CINs [85, 234, 303].

**Fig. 8:**
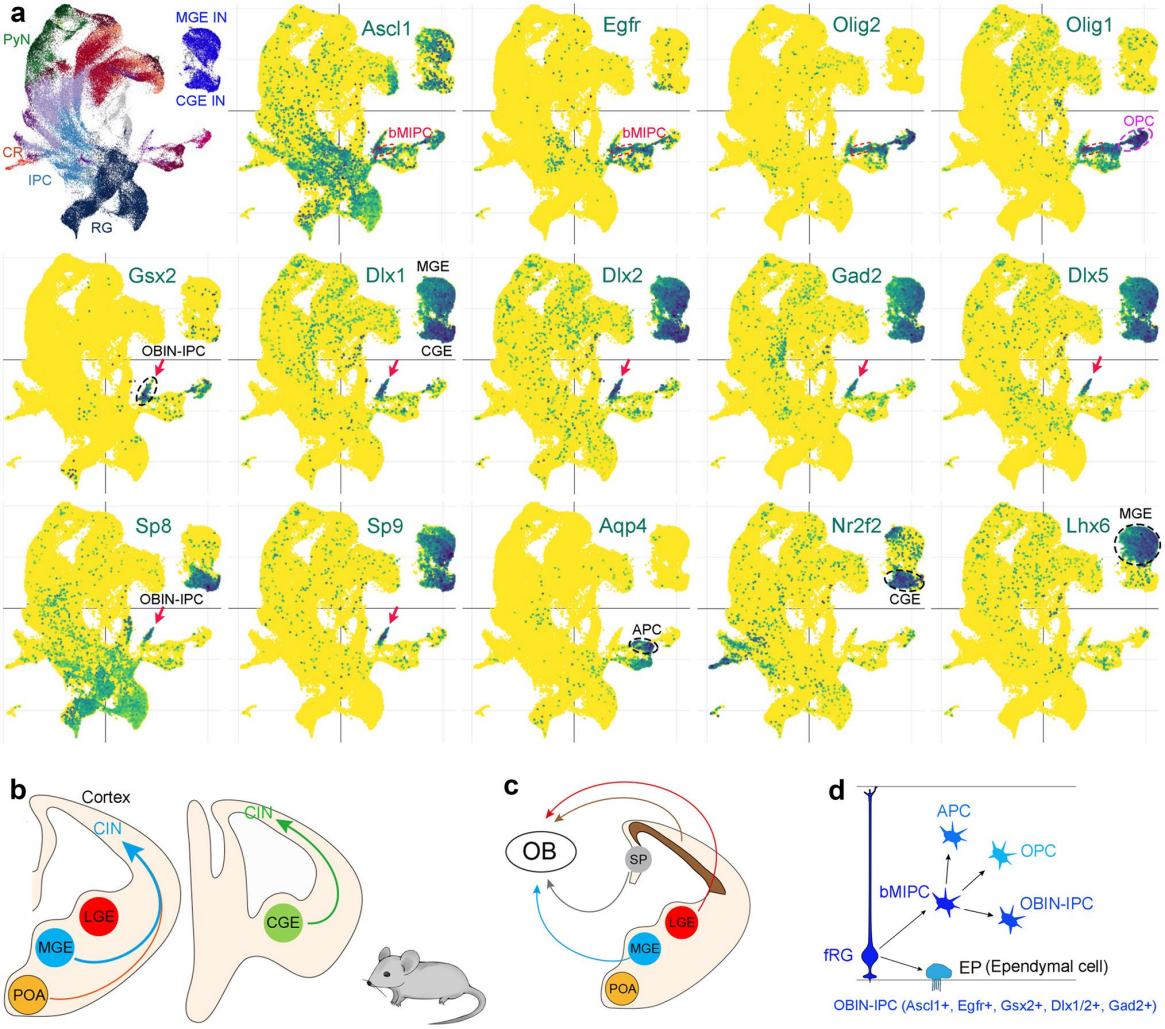
Mouse cortical interneurons (CINs) are derived from subcortical ganglionic eminences (GEs). **a** tSNE plot from scRNA-seq data (a total of 98,047 mouse cortical cells) was obtained using mouse E10.5 to the P4 cortex [306]. Individual cell types are labeled, adapted from Di Bella DJ *et al.*, 2021 [306]. MGE-, POA-, and/or CGE-derived *Dlx1*^+^, and/or *Dlx2*^+^, *Gad2*^+^, *Dlx5*^+^, *Sp8*^+^, *Sp9*^+^, *Nr2f2*^+^, *Lhx6*^+^ CINs are clearly identified. Note that only a small number of IPCs for interneurons that express *Egfr, Gsx2, Dlx1, Dlx2,* and *Gad2* are identified (arrows) adjacent to the bMIPC cluster that express *Ascl1, Egfr, Olig1,* and *Olig2*. Genetic labeling of the bMIPC experiments demonstrates that bMIPCs produce OBINs but not CINs. **b** It is well accepted that mouse CINs are derived from the MGE, POA, and CGE. **c** Mouse OBINs are derived from fRG cells in the developing MGE, LGE, cortex, and septum (SP). After birth, mouse OBINs are derived from B1 NSCs in the SVZ of the lateral ventricle. **d** Mouse cortical fRG cells first give rise to bMIPC, which then give rise to cortical APCs, OPCs, and OBIN-IPCs.

**Fig. 9:**
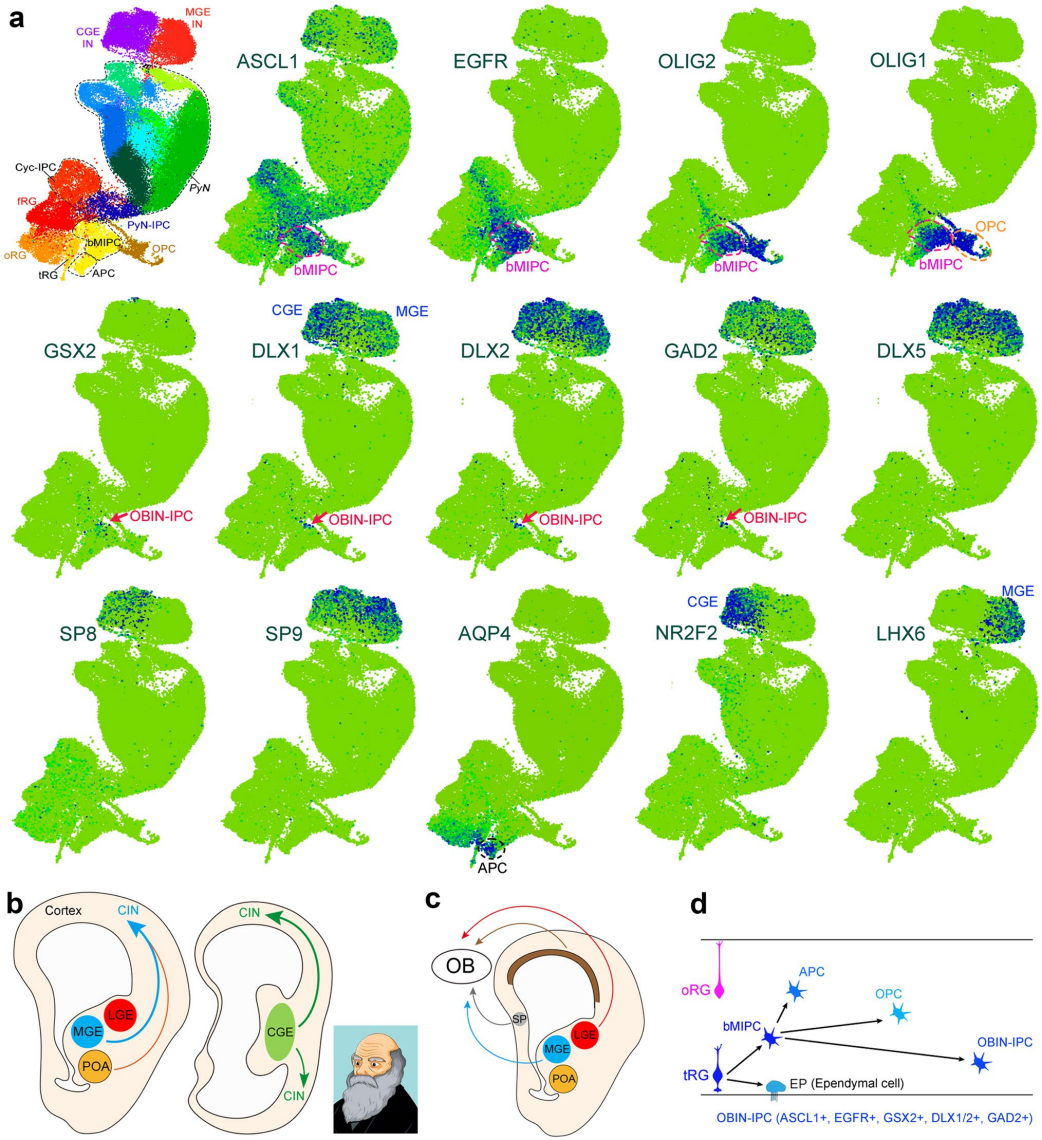
Virtually all human CINs are derived from subcortical ganglionic eminences (GEs), but not from the cortex. **a** tSNE plot from scRNA-seq data (a total of 57,868 human cortical cells) obtained using human GW18 (19,373 cells), GW22 (12,567 cells), GW23 (12,557 cells), and GW26 (13,371 cells) cortical tissue [88]. Individual cell types are labeled, adapted from Trevino *et al.*, 2021 [88]. MGE-, POA-, and/or CGE-derived *DLX1*^+^, and/or *DLX2*^+^, *GAD2*^+^, *DLX5*^+^, *SP8*^+^, *SP9*^+^, *NR2F2*^+^, *LHX6*^+^ CINs are clearly identified. A small number of IPCs for interneurons express *EGFR, GSX2*, *DLX1*, *DLX2*, and *GAD*2 (arrows) are in the bMIPC cluster (*ASCL1*, *EGFR*, *OLIG1*, and *OLIG2)*. This provides evidence that *EGFR*^*+*^*, DLX1*^*+*^*, DLX2*^+^, and *GAD2*^+^ IPCs are OBIN-IPCs, but not CIN-IPCs. Note that *DLX5, SP8, and SP9* expression is not observed in the OBIN-IPC cluster, suggesting that very few immature OBINs are generated by GW23-26. **b** I propose that virtually all human CINs are derived from the MGE, POA, and CGE, similar to mice. **c** I propose that human OBINs are derived from fRG cells in the developing MGE, LGE, cortex, and septum (SP). After birth, human OBIN-IPCs are derived from B1 NSCs in the SVZ of the lateral ventricle. **d** Human cortical tRG cells first give rise to bMIPC, which then give rise to cortical APCs, OPCs, and OBIN-IPCs.

To further evaluate the origins of human CINs, I have re-examined mouse and human cortical scRNA-Seq data and found that nearly all CINs are derived from the subcortical GEs (Fig. 8, 9). Here is the basic logic of this analysis framework. As mentioned above, in mice, CINs appear to be exclusively derived from the subcortical GEs. In humans, if a subset of CINs is derived from cortical RG cells, we should be able to identify IPCs for these CINs in the developing human cortex, and these IPCs must express DLX1 and DLX2 [304, 305]. It is worth noting that a number of IPCs in the human and mouse subcortical GEs express EGFR, but when EGFR-expressing IPCs and their progeny migrate into the cortex, they downregulate EGFR expression [60, 80]. Therefore, all EGFR^+^ cells seen in the mouse and human cortex are produced directly or indirectly from cortical RG cells. I re-analyzed scRNA-seq datasets from a total of 98,047 mouse cortical cells obtained from mouse E10.5 to P4 cortex [306], and a total of 57,868 human cortical cells obtained from human GW18 (19,373 cells), GW22 (12,567 cells), GW23 (12,557 cells), and GW26 (13,371 cells) cortical tissue [88]. I found that, first, it is easy to identify MGE-, POA-, and CGE-derived *DLX1*^+^, and/or *DLX2*^+^, *GAD2*^+^, *DLX5*^+^, *SP8*^+^, *SP9*^+^, *NR2F2*^+^, *LHX6*^+^ CIN clusters in both mice and humans (Fig. 8a, 9a). Second, only a small number of IPCs for interneurons are identified in the mouse and human cortical cells, and they express *EGFR, GSX2, DLX1, DLX2*, and *GAD2* (Fig. 8a, 9a). These IPCs are adjacent to the cortical bMIPC cluster that express *ASCL1, EGFR, OLIG1*, and *OLIG2*, demonstrating that these *GSX2*^*+*^*, DLX1*^*+*^*, DLX2*^*+*^*, GAD2*^+^, and *EGFR*^+^ IPCs are likely to be OBIN-IPCs, but not CIN-IPCs. scRNA-Seq analysis of fluorescence-activated cell sorting EGFR^+^ cells isolated from the frontal lobe of the human cerebral cortex between GW21 and GW26 supported these observations [60, 87]. Immunostaining further validated EGFR^+^ GSX2^+^ cells and EGFR^+^ DLX2^+^ cells in the GW23 cortex [60]. Notably, GSX2^+^ cells in the mouse cortex are bMIPCs that are able to produce cortical APCs, OPCs, and OBIN-IPCs *in vivo* [85], but favor the specification of OBIN identity [307, 308]. Therefore, human cortical tRG cells and mouse cortical fRG cells indeed produce inhibitory interneurons, but these interneurons are for the OB, not for the cortex (Fig. 8b–d, 9b–d). This also strongly suggests that the basic principles of mammalian, including human, cortical development and evolution can be investigated in model systems such as the mouse [54, 55].

## Human-specific Genes Alone May Not Be Essential for Most Cortical Expansion

The human cerebral cortex has 16.3 billion neurons, whereas the chimpanzee cerebral cortex has ~8 billion neurons. This discrepancy has led to extensive studies on human-specific genes over the past 10 years [104, 309–311], as these genes are believed to have significantly contributed to cortical expansion during human evolution. However, I propose that human-specific genes alone might not be fundamental for human cortical expansion.

The chimpanzee cerebral cortex has 8 billion neurons, whereas the macaque cortex has about 1.7 billion neurons. Human-specific genes cannot account for this. The expression of more than 20 human-specific protein-coding genes is enriched in human cortical progenitors [310]. Functional studies of these genes have revealed that many of them can expand cortical RG cells or PyN-IPCs. For example, overexpression of the human-specific gene *ARHGAP11B* in the embryonic mouse neocortex at E13.5 resulted in cortical folding at E18.5 [312]. Furthermore, the *ARHGAP11B*-transgenic marmoset fetal cortex exhibited surface folds at day 101 of the 150-day gestation, in contrast to the smooth surface of the normal control marmoset brain [313]. The authors suggest that *ARHGAP11B* plays a key role in human neocortex expansion. Thus, these human-specific genes may be important in the expansion of the human cortex, alone or in combination. Loss of function analysis in humans, human organoids, and/or experimental primates will be important to show which of these is critical for human cortical expansion. Perhaps some of these genes interact with the ERK-BMP7-GLI3R signaling pathway that I postulate is the core engine driving corticogenesis. Overall, I propose that those genes and signaling pathways that exhibit an evolutionary increase in expression in mammalian cortical RG cells or IPCs are important for cortical development, expansion, and evolution, such as phosphorylated ERK signaling and the *BMP7* gene [54, 55].

## Conclusions and Perspectives

In this review, based on our recent results and those of others, I began by describing the progression of mouse and human cortical RG cell lineages, emphasizing my perspective of their “slow and multistep” changes during the evolution of cortical neurogenesis. Second, I propose that the ERK-BMP7 signaling pathway in mammalian cortical RG cells plays a fundamental role in driving cortical development, expansion, and evolution. Finally, I propose that: (1) SHH signaling is not a key regulator of primate cortical expansion and folding; (2) human cortical radial glial cells do not generate neocortical interneurons; (3) human-specific genes may not be essential for most cortical expansion.

In 1962, Francis Crick said at the time he received the Nobel prize: “Politeness is the poison of all good collaboration in science. The soul of collaboration is perfect candor, and rudeness if need be. A good scientist values criticism almost higher than friendship: no, in science, criticism is the height and measure of friendship” [314]. In this review, I aim to provide clarity on what I believe is correct in the field. Of course, my proposals are hypotheses that should be critically evaluated and experimentally challenged. None-the-less, my fresh perspective merits serious consideration as it provides novel molecular insights into core mechanisms that may underlie how the cortex has expanded during evolution. It is based on evolutionarily old signaling pathways that are tinkered with to achieve increased cortical growth and the generation of a balanced set of cell types.

I have explained why I believe that human-specific genes may not be the central driver for cortical expansion. As shown in Fig. 1, it is clear that there must be a common rule that drives the expansion of the mammalian cortex and brain volume. Obviously, the expansion of the chimpanzee cerebral cortex compared with old-world monkeys has nothing to do with human-specific genes. As pointed out by Francois Jacob: “Evolution is a tinkerer. Evolution works on what already exists, either transforming a system to give it a new function or combining several systems to produce a more complex one” [315]. More and more human-specific genes are being identified. For example, around 900 genes with human-specific features were reported [316]. These human-specific genes may have a function in human brain evolution, but since they are human-specific they cannot be central to how the cortex has grown from rodents to monkeys and then to primates/humans. Other mechanisms must underlie this.

I have also explained why I believe that human cortical oRG cells cannot be versatile NSCs. Previous studies reported that human cortical oRG cells give rise not only to cortical PyNs but also to cortical OPCs and/or CINs in the second trimester [86, 89, 295–297]. It is believed that oRG cells in the OSVZ mainly produce PyNs for the upper cortical layers [56, 57, 60, 61, 98]. Again, as pointed out by Francois Jacob: “Evolution is far from perfection [315], and a new structural feature does not have to be optimal but must be ‘good enough’ to provide a survival advantage for the species” [162, 315]. I believe that the OSVZ cannot provide a niche for almost simultaneously producing cortical PyNs, OPCs, and CINs. In contrast, I believe that human cortical tRG-derived APCs and OPCs have great abilities to proliferate and migrate within the cortex. In other words, there is great evolutionary pressure for oRG cells to generate cortical PyNs, but it is not necessary for oRG cells to produce cortical OPCs and CINs.

As I proposed throughout the review, mammalian cortical evolutionary expansion is most likely driven by the ERK-BMP7-GLI3R signaling pathway in cortical RG cells, which participates in a positive feedback loop through antagonizing SHH signaling. Notably, this positive feedback loop can work like a tinkerer. Because ERK-BMP7-GLI3R signaling and SHH signaling mutually inhibit each other in cortical RG cells, they can drive either cortical evolutionary expansion or cortical evolutionary dwarfism.

And now, for my philosophical perspective. Earth is estimated to exist for at least 1 billion years; this would give us time for ERK-BMP7 signaling is continue driving our brains to evolve and generate a cortex with more and more neurons (Fig. 4). So, perhaps, in the future there will be more intelligent humans living on a peaceful blue planet.
