# Correction to: The Principle of Cortical Development and Evolution

**DOI:** 10.1007/s12264-024-01282-3

**Published:** 2024-08-14

**Authors:** Zhengang Yang

**Affiliations:** State Key Laboratory of Medical Neurobiology and MOE Frontiers Center for Brain Science, Department of Neurology, Institutes of Brain Science, Zhongshan Hospital, Fudan University, Shanghai, 200032 China

**Correction to: Neurosci. Bull.** 10.1007/s12264-024-01259-2

The original version of this article unfortunately contained one error.

The author found that in the legend of Fig. 1, the origin of the figure was missing from this article. The corrected legend should include the following sentence at the end: “The figure is modified from a PowerPoint slide by Professor Wieland B. Huttner, with permission.”

